# Composition–property correlations of SiO_2_ content in CO_2_-based PPC/ionic-liquid composite zinc electrolytes

**DOI:** 10.1039/d6ra08391a

**Published:** 2026-11-11

**Authors:** Guanlin Mu, Shixin An, Guanyu Ren, Haiyue Wang, Liying Guo

**Affiliations:** a Shenyang University of Technology, School of Petrochemical Engineering Liaoyang China lyguo1981@163.com

## Abstract

The performance of multicomponent polymer electrolytes for Zn batteries is governed collectively by the local ionic environment, bulk ion transport, Zn^2+^-associated steady-state response, and electrode/electrolyte interfaces. Here, laboratory-synthesized CO_2_-based poly(propylene carbonate) (PPC) was used as a film-forming matrix, together with PYR_13_TFSI, Zn(TFSI)_2_, and varying contents of SiO_2_, to construct PPC/PYR_13_TFSI/Zn(TFSI)_2_/SiO_2_ solid-state composite polymer electrolytes. Systematic composition–property correlations were established between SiO_2_ content and multiscale properties in a fixed PPC/PYR_13_TFSI/Zn(TFSI)_2_ ternary matrix. Because PPC incorporates CO_2_ as a comonomer-derived carbon source, this material platform provides a CO_2_-valorization-oriented electrolyte design. XRD showed that all samples retained predominantly amorphous polymer matrices, whereas FTIR and peak-fitted O 1s/XPS data revealed composition-dependent changes in the local chemical environments of PPC oxygen-containing groups, TFSI^−^ anions, and Zn-salt species after SiO_2_ incorporation. PIZS-3 (3 wt% SiO_2_) delivered the lowest bulk resistance, a room-temperature ionic conductivity of approximately 6.0 × 10^−4^ S cm^−1^, and the highest apparent Zn^2+^ transference number (0.556). Under stepwise current-density tests at 0.5–5.0 mA cm^−2^ with 1 mAh cm^−2^ per half-cycle, both Zn|PIZ|Zn and Zn|PIZS-3|Zn cells exhibited stable reversible voltage profiles. The Zn|PIZS-3|Zn symmetric cell operated stably for more than 1200 h at 1 mA cm^−2^ and 1 mAh cm^−2^ per half-cycle (1 h per half-cycle), evidencing robust Zn–metal interfacial behavior. The V_2_O_5_|PIZS-3|Zn full cell also exhibited excellent cycling stability, whereas PIZS-7 delivered the highest rate capacity within the tested SiO_2_ range of 0–7 wt%. These results identify *ca.* 3 wt% SiO_2_ as an optimal composition window for balancing bulk transport and Zn–metal interfacial response, while higher SiO_2_ contents favor rate capability in full cells.

## Abstract 2

SiO_2_ content tunes CO_2_-derived PPC composite zinc electrolytes. PIZS-3 (3 wt%) optimizes ionic transport and Zn–metal stability, whereas PIZS-7 delivers the highest full-cell rate capacity.

## Graphical abstract

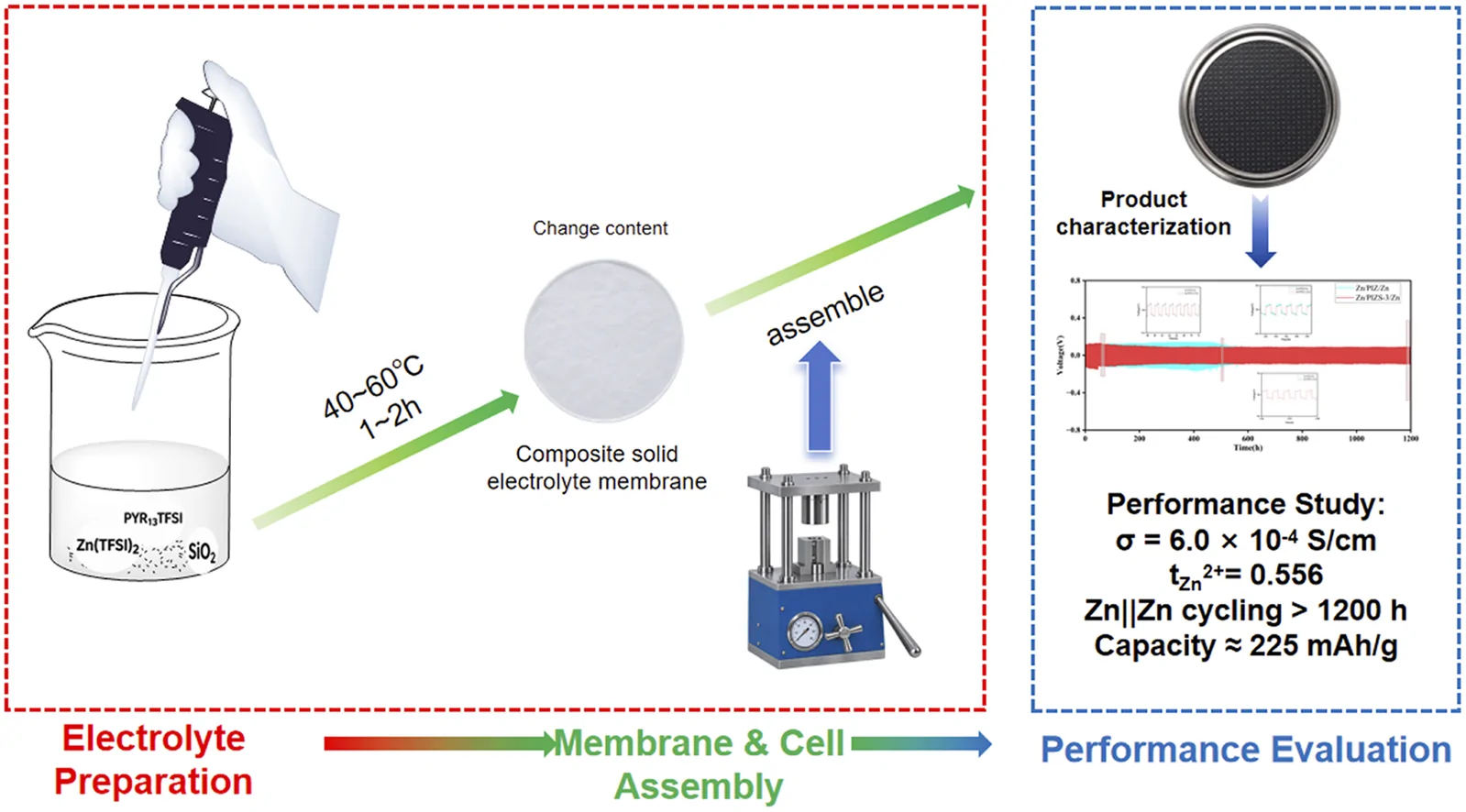

## 1 Introduction

Rechargeable Zn–metal batteries are attractive for large-scale and intrinsically safe energy-storage applications because of the earth abundance of Zn, controllable cost, and high theoretical capacity of the Zn–metal anode.^1–4^ Polymer solid-state electrolytes can improve battery safety, enhance electrode/electrolyte contact, and regulate Zn plating/stripping by establishing continuous ion-transport phases.^1–3,34,37^ The design of high-performance polymer electrolytes for Zn batteries therefore focuses on bulk ion transport, Zn^2+^-associated migration, and Zn–metal interfacial stability. In multicarrier systems, total ionic conductivity reflects the combined transport of all mobile species, whereas Zn^2+^-associated transport is regulated by anion mobility, ionic association, and local coordination states. Accordingly, bulk conductivity, Zn^2+^-associated migration, and Zn–metal interfacial behavior are key dimensions for evaluating composition-dependent electrolyte properties.

Poly(propylene carbonate) (PPC) is an aliphatic polycarbonate produced by copolymerization of CO_2_ and propylene oxide. Its backbone is rich in carbonyl and carbonate-oxygen sites and exhibits good film-forming ability, making PPC a suitable matrix for CO_2_-based polymer electrolytes. From the perspective of green chemistry and carbon-resource utilization, incorporating CO_2_ as a comonomer-derived carbon source in PPC provides a CO_2_-valorization-oriented material platform for energy-storage applications. PPC-based solid-state electrolytes provide a polar polymer environment for salt dispersion and ion migration and can form continuous ion-transport phases with ionic components.^5,6^ Studies of PPC electrolytes in lithium systems have established an important basis for understanding film formation, polymer–ion interactions, and local-structure regulation. In Zn-salt systems, interactions of Zn^2+^ with polymer coordination sites and anions further determine the cation-transport contribution; suppressing anion migration and regulating local polymer mobility can effectively enhance Zn^2+^-associated transport.^7,8^

The incorporation of PYR_13_TFSI into PPC creates an ion-rich transport environment with enhanced local mobility. The PPC/PYR_13_TFSI/Zn(TFSI)_2_ system studied here contains PPC oxygen-containing sites, Zn^2+^, TFSI^−^, and PYR_13_^+^. Its macroscopic ion transport is governed cooperatively by polymer-segmental motion, Zn^2+^ coordination, TFSI^−^-associated ionic association, and migration of ionic-liquid components. Increasing salt content, optimizing the local polymer environment, and constructing selectively cation-conducting pathways can substantially improve total ionic conductivity, the Zn^2+^ transport contribution, and Zn–metal interfacial stability.^9–13,33,40^

Introducing inorganic nanophases into a polymer/salt/ionic-liquid multicomponent environment creates polymer/inorganic heterointerfaces and provides an additional means of regulating the local ionic environment. Inorganic-filler surfaces can interact with Zn^2+^, TFSI^−^, and polymer chains, thereby modulating local coordination and ion-transport behavior.^14,32,36,38,39^ Such nanofillers therefore connect surface chemistry, local polymer structure, and Zn^2+^ transport, offering an effective route to optimize multicomponent polymer electrolytes.

Fumed SiO_2_ has a high specific surface area and abundant surface oxygen-containing sites, enabling the formation of numerous polymer/inorganic interfaces within a polymer matrix. Previous studies of Zn-based composite polymer electrolytes have shown that SiO_2_ can regulate electrolyte-network structure and the local ion-transport environment.^15,29,31,32,35^ In the PPC/PYR_13_TFSI/Zn(TFSI)_2_ ternary matrix, hydrophilic fumed SiO_2_ promotes cooperative interactions with PPC oxygen-containing groups, Zn salt, and TFSI^−^, thereby regulating the ionic environment adjacent to polymer/inorganic interfaces. As SiO_2_ content changes, the number of inorganic interfaces, local segmental constraints, and the effective transport environment in the continuous polymer phase evolve concurrently. SiO_2_ content is thus a key compositional variable linking local chemical environments to macroscopic ion transport.

SiO_2_ content substantially affects bulk ion transport, the Zn–metal interface, and full-cell response. Local Zn^2+^ coordination, anion participation, and the organic/inorganic interfacial environment collectively regulate Zn plating/stripping and interfacial stability.^16–18,32,34,36^ Full-cell performance is additionally influenced by cathode-reaction kinetics, transmembrane ion transport, and interfacial processes at both electrodes.^19,20^ Different evaluation scales can therefore favor different SiO_2_ contents. Establishing systematic relationships between SiO_2_ content, bulk transport, the Zn–metal interface, and full-cell response enables selection of an optimal composition window for a defined function.

In this work, laboratory-synthesized CO_2_-based PPC was employed as the polymer matrix, while PYR_13_TFSI and Zn(TFSI)_2_ were used to construct a PPC/PYR_13_TFSI/Zn(TFSI)_2_ ternary ion-transport matrix. Different contents of SiO_2_ were then introduced to form solid-state composite polymer electrolytes. Component-reference samples were first used to resolve the structural and spectroscopic responses associated with PPC, ionic liquid, Zn salt, and SiO_2_ incorporation. Subsequently, with PPC/PYR_13_TFSI/Zn(TFSI)_2_ fixed at 60 : 20 : 20, the PIZ/PIZS-*x* series was used to establish correlations between SiO_2_ content and bulk transport, Zn–metal interfacial behavior, and full-cell response. XRD, FTIR, and XPS were used to characterize long-range structure and local chemical environments; EIS, temperature-dependent ionic conductivity, and dc polarization were used to evaluate bulk and Zn^2+^-associated transport; Zn|Zn symmetric cells were used to assess Zn plating/stripping interfacial behavior; and V_2_O_5_|electrolyte|Zn full cells were used to evaluate rate and cycling performance across compositions. This work also demonstrates the CO_2_-valorization potential of PPC in solid-state zinc electrolytes.

## 2 Experimental section

### 2.1 Materials

PPC was synthesized in our laboratory through the alternating copolymerization of CO_2_ and propylene oxide, following sustainable PPC synthesis strategies reported recently.^20^. (CAS No. 25511-85-7) and had a number-average molecular weight of approximately 50 000. The number-average molecular weight was determined by gel-permeation chromatography (GPC; PL-GPC50). PPC was vacuum-dried at 50 °C for 24 h before use.

1-Methyl-1-propylpyrrolidinium bis(trifluoromethanesulfonyl)imide (PYR_13_TFSI; CAS No. 223437-05-6; 98.0%) was purchased from Macklin (product no. M814165-25 g) and vacuum-dried before use.

Zinc bis(trifluoromethanesulfonyl)imide [Zn(TFSI)_2_; CAS No. 168106-25-0; 95%] was purchased from Macklin (product no. Z769214-25 g) and vacuum-dried at 80 °C for 24 h before use.

Hydrophilic fumed nanosilica (hydrophilic-150, SiO_2_; CAS No. 60676-86-0; 99.8%; BET specific surface area: 150 m^2^ g^−1^; particle size: 7–40 nm) was purchased from Macklin (product no. N817579-100 g) and vacuum-dried before use.

Anhydrous acetonitrile was used as the solvent. Unless otherwise specified, all reagents were stored in sealed containers after use, and exposure to atmospheric moisture was minimized during handling of Zn(TFSI)_2_, PYR_13_TFSI, and preparation of the composite electrolytes.

### 2.2 Preparation of composite electrolytes and sample nomenclature

PPC-based composite polymer electrolyte membranes were prepared by solution casting. PPC was first dissolved in anhydrous acetonitrile under magnetic stirring at 40 °C to obtain a homogeneous transparent solution. PYR_13_TFSI and/or Zn(TFSI)_2_ were then added according to the designated composition, followed by further stirring until a homogeneous mixture was obtained. For SiO_2_-containing samples, SiO_2_ was first dispersed in a small amount of anhydrous acetonitrile and ultrasonicated for 30 min before addition to the polymer solution to improve filler dispersion in the PPC matrix.

The casting solution was poured into a polytetrafluoroethylene mold and the solvent was allowed to evaporate slowly at room temperature for 8 h. The resulting film was then vacuum-dried at 40 °C for 4 h and subsequently at 60 °C for 12 h. The membrane diameter was approximately 18 mm. Electrolyte membranes used in symmetric and full cells were controlled to a thickness below 40 µm and stored under identical conditions. For ionic-conductivity calculations, the thickness of each membrane was measured individually before cell assembly and the measured value for that sample was used. The thickness variation measured at different positions on the same membrane did not exceed 3 µm, within preparation and measurement uncertainty. The resulting free-standing membranes served directly as electrolyte separators, and no additional free-flowing liquid electrolyte was introduced during testing. Because the PYR_13_TFSI was confined within the PPC matrix, the materials are consistently referred to here as solid-state composite polymer electrolytes.

Dispersion reproducibility was assessed by SEM after the 30 min ultrasonication procedure. A representative overview image showed no obvious macroscopic SiO_2_-rich region within the observed field of view. Two additional SEM images obtained from independently prepared samples showed similar particle/aggregate morphology and comparable spatial distributions, providing qualitative evidence for preparation reproducibility. Because these were secondary-electron SEM images, they were used as morphological evidence of SiO_2_ dispersion reproducibility.

Before cell assembly, membrane thickness was measured at five positions on each membrane. All membranes used for electrochemical testing were thinner than 40 µm, and the maximum thickness difference between positions on the same membrane was within 3 µm. The thickness statistics report the mean thickness, standard deviation, number of independently prepared membranes (*n*), and the definition of the error bars; all error bars represent mean ± SD. The measured values were PIZ: 32.4 ± 1.1 µm (maximum in-plane difference 2.5 µm, *n* = 5); PIZS-1: 32.0 ± 1.0 µm (2.3 µm, *n* = 5); PIZS-3: 31.8 ± 0.9 µm (2.1 µm, *n* = 5); PIZS-5: 32.2 ± 1.0 µm (2.4 µm, *n* = 5); PIZS-7: 32.7 ± 1.1 µm (2.6 µm, *n* = 5); and PIZS-9: 33.3 ± 1.2 µm (2.8 µm, *n* = 5).

The proportions of PPC, PYR_13_TFSI, and Zn(TFSI)_2_ are expressed as feed mass parts. The SiO_2_ loading is expressed as a mass percentage relative to the total mass of the corresponding PIZ ternary matrix. For example, PIZS-3 denotes the electrolyte obtained by adding 3 wt% SiO_2_ to a PPC/PYR_13_TFSI/Zn(TFSI)_2_ = 60 : 20 : 20 ternary matrix.

Sample nomenclature comprises two groups. The component-reference series in Table 1 was used to identify structural and spectroscopic changes arising from different component combinations. The PIZ/PIZS-*x* series in Table 2 was used to establish relationships between SiO_2_ content and transport/electrochemical response. PIZ is used exclusively for the SiO_2_-free 60 : 20 : 20 ternary matrix, whereas *x* = 1, 3, 5, 7, and 9 in PIZS-*x* denotes the additional SiO_2_ content in wt%.

**Table 1:** Compositions of component-reference samples

Sample	Composition	Feed ratio
PPC	PPC	100
PI-40	PPC + PYR_13_TFSI	60 : 40
PZ-40	PPC + Zn(TFSI)_2_	60 : 40
PS-3	PPC + SiO_2_	100 + 3 wt% SiO_2_
PIS-3	PPC + PYR_13_TFSI + SiO_2_	60 : 40 + 3 wt% SiO_2_
PZS-3	PPC + Zn(TFSI)_2_ + SiO_2_	60 : 40 + 3 wt% SiO_2_
PIZ	PPC + PYR_13_TFSI + Zn(TFSI)_2_	60 : 20 : 20
PIZS-3	PPC + PYR_13_TFSI + Zn(TFSI)_2_ + SiO_2_	60 : 20 : 20 + 3 wt% SiO_2_

**Table 2:** Compositions of PIZS-*x* series composite electrolytes

Sample	Composition	Feed ratio
PIZ	PPC + PYR_13_TFSI + Zn(TFSI)_2_	60 : 20 : 20
PIZS-1	PPC + PYR_13_TFSI + Zn(TFSI)_2_ + SiO_2_	60 : 20 : 20 + 1 wt% SiO_2_
PIZS-3	PPC + PYR_13_TFSI + Zn(TFSI)_2_ + SiO_2_	60 : 20 : 20 + 3 wt% SiO_2_
PIZS-5	PPC + PYR_13_TFSI + Zn(TFSI)_2_ + SiO_2_	60 : 20 : 20 + 5 wt% SiO_2_
PIZS-7	PPC + PYR_13_TFSI + Zn(TFSI)_2_ + SiO_2_	60 : 20 : 20 + 7 wt% SiO_2_
PIZS-9	PPC + PYR_13_TFSI + Zn(TFSI)_2_ + SiO_2_	60 : 20 : 20 + 9 wt% SiO_2_

PPC, PI-40, PZ-40, PS-3, PIS-3, PZS-3, and PIZ were used primarily as component-reference samples. PIZS-3 served both as the complete quaternary sample in the component-reference series and as a member of the PIZS-*x* main series. Throughout this work, the PIZ/PIZS-*x* nomenclature is used when discussing the effect of SiO_2_ content.

With PPC/PYR_13_TFSI/Zn(TFSI)_2_ fixed at 60 : 20 : 20, PIZS-1, PIZS-3, PIZS-5, PIZS-7, and PIZS-9 were further prepared to compare the effects of SiO_2_ content on bulk transport and electrochemical response; their formulations are listed in Table 2. PIZS-9 was included to extend the EIS composition gradient and confirm the impedance trend at high filler content. Because its room-temperature impedance had already increased, subsequent dc-polarization and full-cell tests focused on PIZS-1, PIZS-3, PIZS-5, and PIZS-7. Accordingly, conclusions concerning Zn^2+^-associated steady-state response and full-cell performance are restricted to the corresponding tested composition range.

### 2.3 Structural and local chemical environment characterization

X-ray diffraction (XRD; Bruker D8 Advance) was used to analyze the phase characteristics of the electrolyte membranes over 2*θ* = 5–90° at a scan rate of 5° min^−1^. Fourier-transform infrared spectroscopy (FTIR; Thermo Nicolet IS5) was used to compare the vibrational environments of C=O, C–O, and TFSI^−^-related groups in PPC over 4000–500 cm^−1^. X-ray photoelectron spectroscopy (XPS; Thermo ESCALAB 250Xi) was used to compare surface elemental composition and core-level responses; binding energies were calibrated to C 1s = 284.8 eV. All XPS binding energies cited in the text are rounded to one decimal place. To examine changes in PPC oxygen-containing groups, the O 1s envelope was deconvoluted into components associated with C=O and C–O environments; an Si–O-related contribution was additionally considered for the SiO_2_-containing electrolyte. The fitted spectra are shown in Fig. 4. The main text focuses on the Zn 2p, N 1s, and Si 2p signals. To avoid overinterpretation of small peak shifts, XPS results were assessed together with the corresponding FTIR changes.

### 2.4 Electrochemical characterization

The ionic conductivities of the composite electrolyte membranes were determined by electrochemical impedance spectroscopy (EIS). Both EIS and dc polarization were conducted on a CHI760F electrochemical workstation. Electrolyte membranes were sandwiched between two stainless-steel electrodes to form SS|electrolyte|SS blocking cells and measured over a frequency range of 10^6^–0.01 Hz. Ionic conductivity was calculated according to eqn (1):^21^1
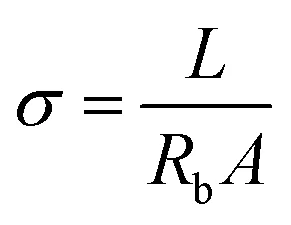
where *σ* is the ionic conductivity (S cm^−1^), *L* is the measured thickness of an individual electrolyte membrane (cm), *A* is the effective overlap area between the stainless-steel electrode and electrolyte membrane (cm^2^), and *R*_b_ is the bulk resistance (Ω) obtained from the high-frequency EIS response. The ionic conductivity of each sample was calculated using its own *L*, *A*, and *R*_b_ values. To compare thermally activated transport over a broader temperature range, temperature-dependent EIS measurements were performed from 20 to 80 °C; impedance spectra were recorded and the corresponding ionic conductivities were calculated after stabilization at each set temperature.

The apparent Zn^2+^ transference number was estimated from dc polarization combined with EIS using Zn|electrolyte|Zn symmetric cells. Before polarization, EIS was performed to obtain the initial interfacial resistance (*R*_0_). A constant dc polarization voltage (Δ*V*) was then applied and the initial current (*I*_0_) and steady-state current (*I*_s_) were recorded. After polarization, EIS was performed again to obtain the post-polarization interfacial resistance (*R*_s_). The apparent Zn^2+^ transference number was calculated according to eqn (2):^22^2
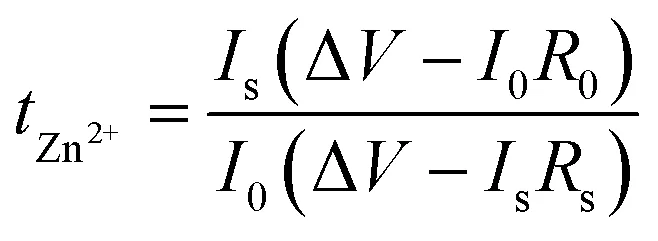
where *t*_Zn^2+^_ is the apparent Zn^2+^ transference number, Δ*V* is the polarization voltage, *I*_0_ and *I*_s_ are the initial and steady-state currents, respectively, and *R*_0_ and *R*_s_ are the interfacial resistances before and after polarization, respectively. Because the system contains multiple mobile species, including Zn^2+^, TFSI^−^, and PYR_13_^+^, this value is used as an apparent metric for comparing Zn^2+^-associated polarization responses under identical test conditions.

To evaluate the interfacial response between the composite electrolytes and Zn–metal anodes, Zn|electrolyte|Zn symmetric cells were assembled and subjected to galvanostatic Zn plating/stripping on a CT-4008Tn-mA battery tester. Long-term cycling was performed at 1 mA cm^−2^ with an areal capacity of 1 mAh cm^−2^ per half-cycle. Based on *Q* = *j* × *t*, each half-cycle lasted 1 h. Zn|PIZ|Zn and Zn|PIZS-3|Zn symmetric cells were also tested at 30 °C using a stepped current-density sequence of 0.5, 1, 2, 3, 5, 3, 2, 1, and 0.5 mA cm^−2^, with 1 mAh cm^−2^ per half-cycle at each step. Voltage profiles were recorded throughout the tests to compare Zn plating/stripping polarization and interfacial response among the composite electrolytes.

Full-cell performance was evaluated using V_2_O_5_|electrolyte|Zn cells, in which V_2_O_5_ served as the cathode active material, Zn foil as the anode, and the electrolyte-membrane thickness was controlled below 40 µm. The thickness variation measured at different positions on the same membrane did not exceed 3 µm. The mass ratio of active material, conductive additive, and binder in the V_2_O_5_ cathode was 7 : 2 : 1, and the active-material loading was approximately 0.71 mg cm^−2^. Galvanostatic charge/discharge testing was conducted on a CT-4008Tn-mA battery tester using a 1.5 V voltage window. Rate performance was evaluated sequentially at 0.1, 0.2, 0.5, 1, and 2 C, followed by a return to 0.1 C to assess capacity recovery. Cycling stability was evaluated using the prescribed long-term cycling protocol. All specific discharge capacities were calculated based on the mass of active V_2_O_5_ in the cathode.

The V_2_O_5_ loading used here (approximately 0.71 mg cm^−2^) is relatively low compared with practical cathode loadings. This loading was selected to reduce electrode-thickness-related transport limitations and to enable a clearer comparison of electrolyte-dependent rate behavior. Accordingly, the full-cell data should be interpreted primarily as a comparative assessment of electrolyte/cathode compatibility and rate response, rather than as a demonstration of practical high-areal-capacity operation.

## 3 Results and discussion

### 3.1 Amorphous structural basis and evolution of the local chemical environment

To distinguish changes in long-range phase characteristics from those in the local chemical environment, XRD, FTIR, and XPS were first used to analyze the structural response following component incorporation. XRD was used to determine whether composite formation induced new long-range ordered phases, whereas FTIR and XPS were used to track changes in local interactions among PPC oxygen-containing groups, TFSI^−^, Zn salt, and SiO_2_ surface sites.

As shown in Fig. 1, pristine PPC exhibits a pronounced broad diffuse peak at approximately 20–25°, together with a broad scattering background at higher angles, characteristic of a predominantly amorphous structure with short-range order. After PYR_13_TFSI incorporation, the position and profile of the main broad peak changed. Further addition of Zn(TFSI)_2_ and SiO_2_ retained broad-feature-dominated patterns without new sharp reflections from crystalline salts. The different components primarily changed the peak position, broadening, and relative intensity of the diffuse features rather than generating resolvable long-range crystalline phases. The PPC-rich amorphous environment was therefore retained throughout composite formation, and the subsequent transport differences are primarily associated with changes in short-range packing and local ionic interactions.^16,25^

**Fig. 1:**
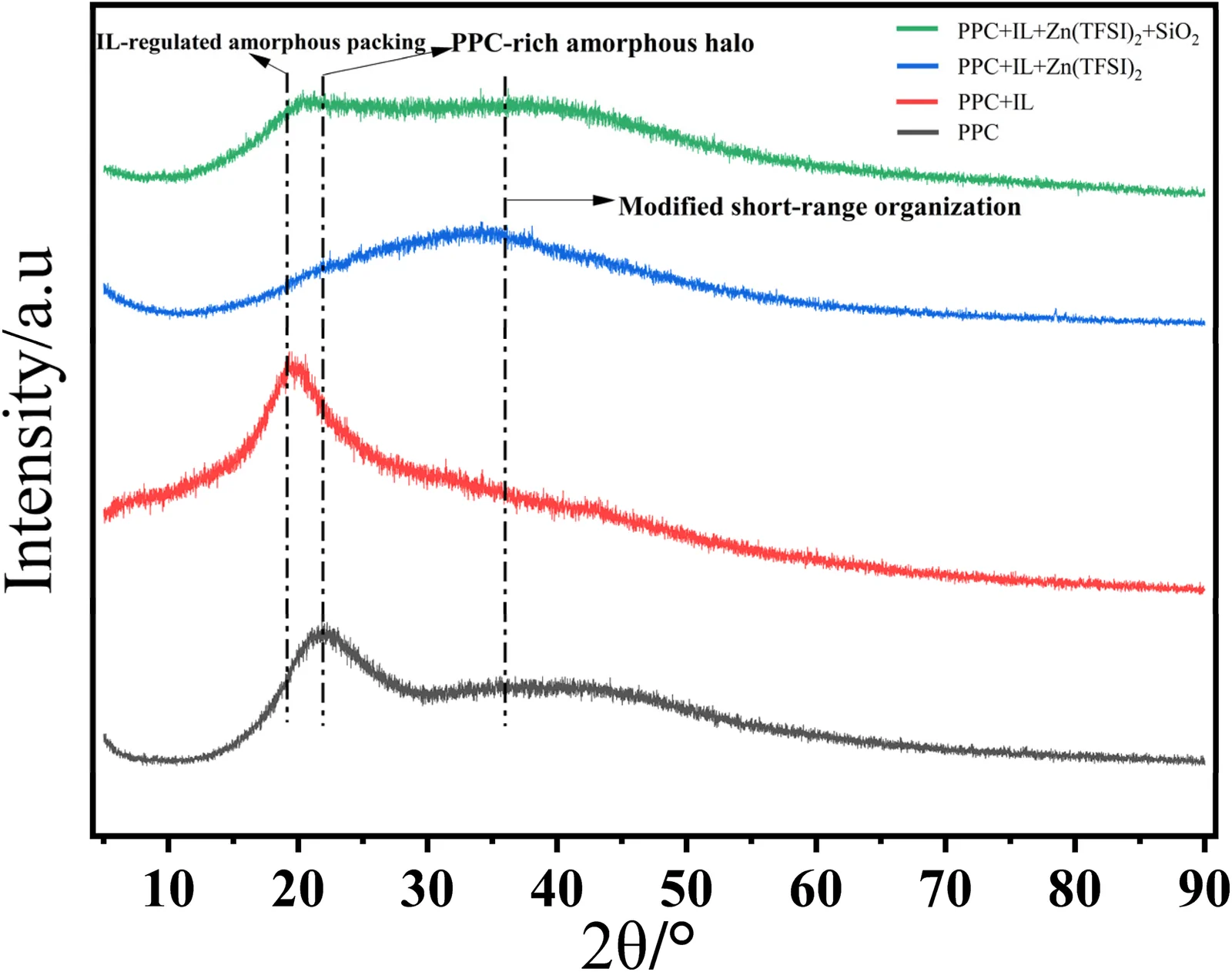
XRD patterns of PPC, PPC + IL, PPC + IL + Zn(TFSI)_2_, and PPC + IL + Zn(TFSI)_2_ + SiO_2_ composite electrolytes. The corresponding compositions (PPC : PYR_13_TFSI : Zn(TFSI)_2_) are PPC (100 : 0 : 0), PPC + IL (60 : 40 : 0), PPC + IL + Zn(TFSI)_2_ (60 : 20 : 20), and PPC + IL + Zn(TFSI)_2_ + SiO_2_ (60 : 20 : 20 + 3 wt% SiO_2_), respectively. Data were acquired over 2*θ* = 5–90° at 5° min^−1^.

Within this common amorphous structural background, FTIR further resolves changes in the vibrational environments of oxygen-containing groups and TFSI^−^.

Fig. 2a shows that all composite samples retain the principal vibrational features of the PPC carbonate backbone, indicating that incorporation of ionic liquid, Zn salt, and SiO_2_ does not alter the primary chemical framework of PPC. Local magnification reveals clearer composition-dependent responses. The C=O absorption band shifts within approximately 1739.5–1743.9 cm^−1^ across the compositions. PPC and PPC + SiO_2_ show similar peak positions, whereas Zn-salt-containing samples and the complete quaternary system display more pronounced changes in the C=O region. Concurrent changes are also observed in the C–O/TFSI^−^ region at 1300–1000 cm^−1^ and in the Si–O/TFSI^−^-related low-wavenumber region at 1000–500 cm^−1^.

**Fig. 2:**
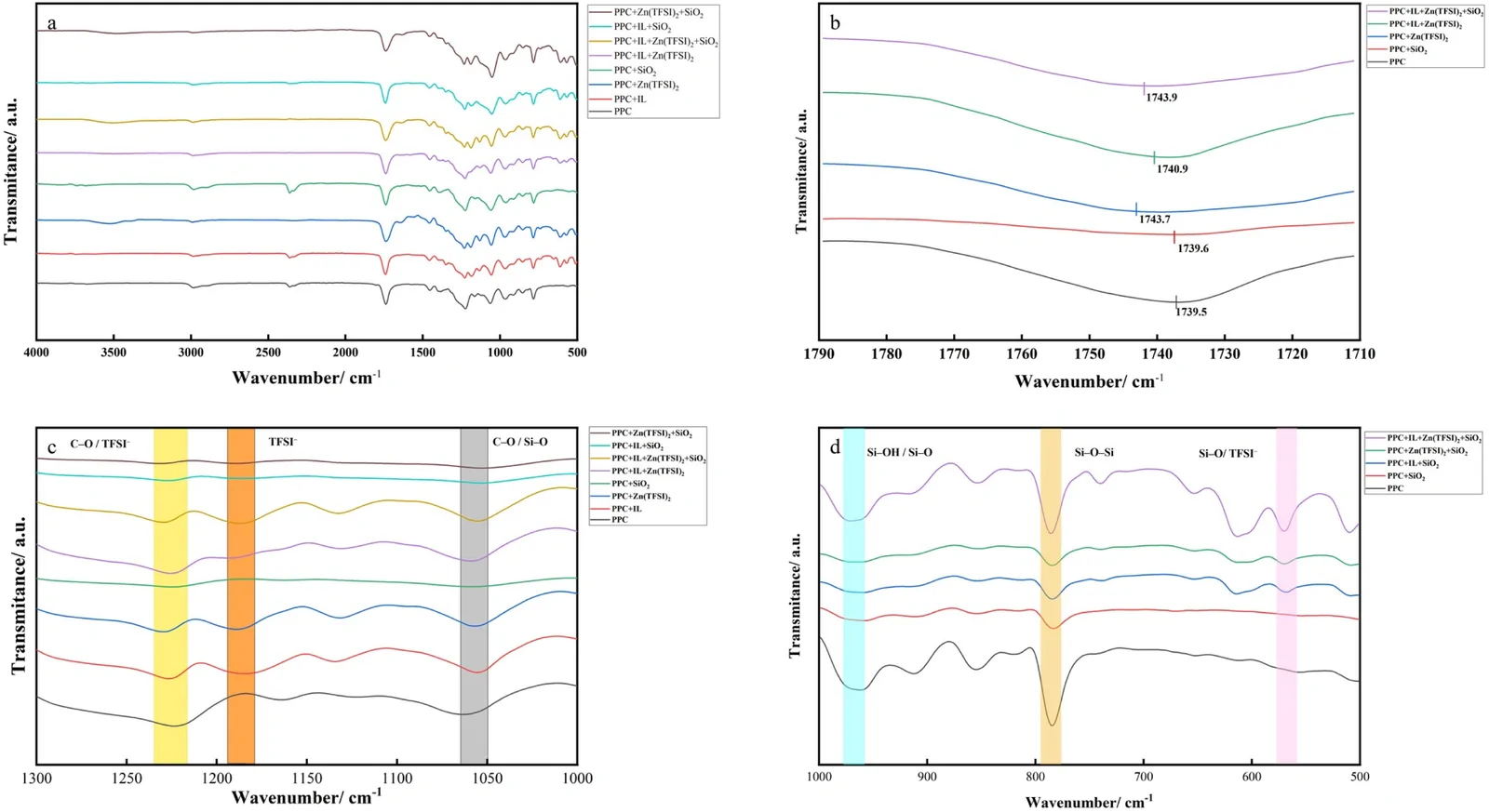
FTIR spectra of PPC-based composite electrolytes: (a) full spectra; (b) C=O region; (c) C–O/TFSI^−^-related region; and (d) low-wavenumber Si–O/TFSI^−^-related region.

These changes are concentrated in regions associated with PPC oxygen-containing groups, TFSI^−^ vibrations, and SiO_2_, indicating that SiO_2_ incorporation is coupled to changes in local interactions within the PPC/ionic-liquid/Zn-salt system. Notably, SiO_2_ addition to PPC alone produces only a minor C=O shift, whereas stronger responses occur in multicomponent systems containing both Zn salt and ionic liquid. This result demonstrates that the effect of the inorganic surface is coupled with the pre-existing ionic environment.^12,13,16,23,24^

XPS was then used to examine this change in the local environment from an electronic-structure perspective. The O 1s envelopes of PPC, PPC + IL, PPC + IL + Zn(TFSI)_2_, and PPC + IL + Zn(TFSI)_2_ + SiO_2_ were peak-fitted to resolve component-dependent changes in oxygen-containing environments. Fig. 3 focuses on the Zn 2p, N 1s, and Si 2p regions, and the corresponding O 1s, Zn 2p, and N 1s peak fitting is provided in Fig. 4.

**Fig. 3:**
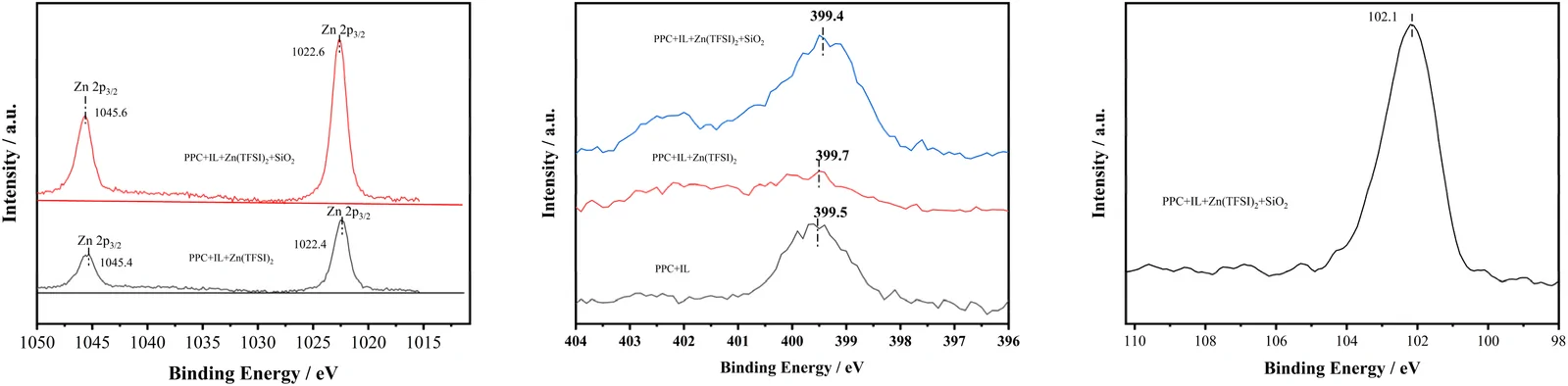
XPS spectra of PPC-based composite electrolytes in the Zn 2p, N 1s, and Si 2p regions.

**Fig. 4:**
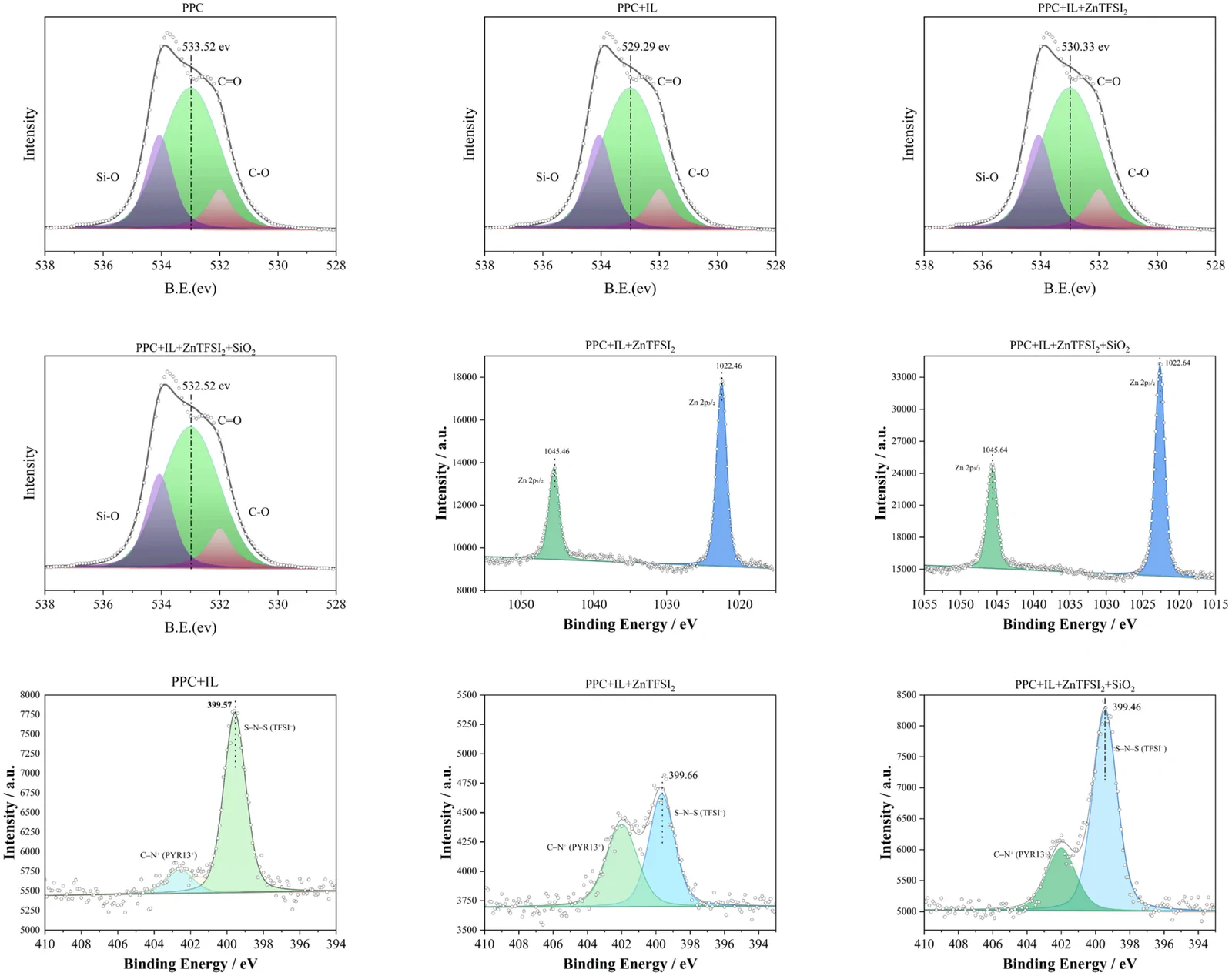
Peak-fitted XPS spectra of O 1s for PPC, PPC + IL, PPC + IL + Zn(TFSI)_2_, and PPC + IL + Zn(TFSI)_2_ + SiO_2_, together with fitted Zn 2p and N 1s regions of the selected composite electrolytes.

The fitted O 1s spectra resolve PPC-derived C=O and C–O-related components and include an Si–O-related contribution for the SiO_2_-containing electrolyte. Across the sequential incorporation of ionic liquid, Zn salt, and SiO_2_, the positions and relative envelopes of the PPC-derived oxygen components evolve, indicating a composition-dependent oxygen-containing coordination environment. The stronger O 1s/FTIR response in the complete quaternary system than in PPC + SiO_2_ alone further indicates that the SiO_2_ surface effect is coupled to the pre-existing ionic environment rather than arising from simple filler addition. After SiO_2_ incorporation, the Zn 2p_3/2_ and Zn 2p_1/2_ peaks shift from approximately 1022.4 and 1045.4 eV to approximately 1022.6 and 1045.6 eV, respectively. The N 1s features also evolve with the sequential incorporation of ionic liquid, Zn salt, and SiO_2_. In addition, the quaternary system shows a distinct Si 2p signal at approximately 102.2 eV. Together, the O-, Zn-, N-, and Si-related changes are consistent with SiO_2_-induced reorganization of the local chemical environment around Zn(TFSI)_2_/TFSI^−^ and PPC oxygen-containing sites.^12,16–18,23,24^

Taken together, XRD, FTIR, and peak-fitted O 1s/XPS data show that the composite systems retain the amorphous character of the PPC matrix, while SiO_2_ incorporation is associated with changes in the local chemical environments of oxygen-containing groups, TFSI^−^, and Zn salt. The component-reference results therefore support an interfacial-modifier role for SiO_2_, in which the inorganic surface participates in local coordination and ion-transport-environment regulation within the multicomponent matrix. Because the O 1s components overlap and XPS is surface sensitive, these data do not uniquely establish a single coordination geometry or interfacial structure; instead, the proposed mechanism is supported by the combined O 1s, Zn/N/Si XPS, FTIR, and electrochemical evidence. The following PIZ/PIZS-*x* series, based on a fixed ternary matrix, further links this local regulation to SiO_2_-content-dependent transport and electrochemical properties.

### 3.2 Effects of SiO_2_ content on bulk ion transport and Zn^2+^-associated migration

Varying SiO_2_ content in a fixed PPC/PYR_13_TFSI/Zn(TFSI)_2_ = 60 : 20 : 20 ternary matrix enables examination of the relationship between polymer/inorganic interfacial composition and bulk-transport response.

Fig. 5 shows that the impedance response of PIZS-1, PIZS-3, PIZS-5, PIZS-7, and PIZS-9 does not vary monotonically with SiO_2_ content. Increasing the SiO_2_ content from 1 to 3 wt% markedly decreases the impedance, whereas further increases to 5–9 wt% raise the impedance again. PIZS-3 exhibits the high-frequency response closest to the origin and lies in the lowest-impedance region. Its corresponding room-temperature ionic conductivity is approximately 6.0 × 10^−4^ S cm^−1^. These results establish an optimal composition window for SiO_2_-mediated ion transport rather than a simple filler-content/conductivity relationship.

**Fig. 5:**
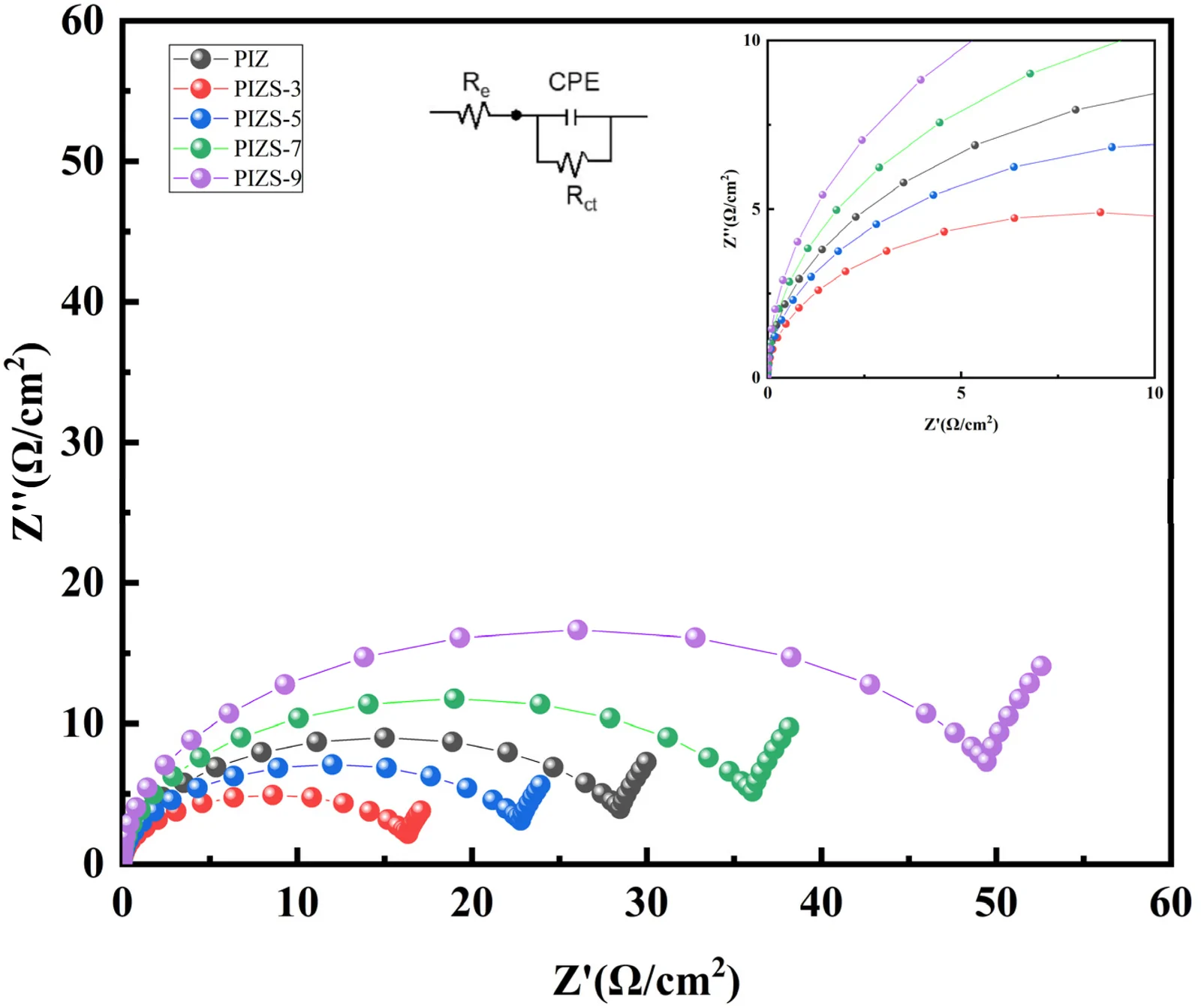
Room-temperature Nyquist plots of PIZ and PIZS-*x* composite electrolytes in SS|electrolyte|SS cells, where (*x*) denotes the SiO_2_ content. The upper-right inset shows the enlarged high-frequency region, while the upper-middle inset presents the equivalent circuit used for EIS fitting, consisting of (*R*_e_) in series with a parallel (*R*_ct_)-CPE element. EIS spectra were acquired over 10^6^–0.01 Hz.

Direct inspection of Fig. 5 shows that the impedance of the PIZS-*x* series decreases as SiO_2_ content increases from 1 to 3 wt% and increases again when the content is raised to 5–9 wt%. PIZS-3 lies in the lowest-impedance region and exhibits the highest apparent Zn^2+^ transference number among the tested PIZS-1, PIZS-3, PIZS-5, and PIZS-7 samples. PIZS-9 was included only to extend the EIS composition gradient and was not subjected to dc-polarization or full-cell testing; therefore, its Zn^2+^-associated steady-state response and full-cell performance are not compared here.

The nonmonotonic impedance trend is consistent with the local spectroscopic responses of the component-reference samples, indicating that SiO_2_ content regulates local ionic interactions and bulk transport through polymer/inorganic interfaces. A moderate SiO_2_ content optimizes the local ionic environment while retaining a continuous polymer-transport phase, allowing PIZS-3 to achieve the lowest impedance. At higher SiO_2_ contents, increased polymer/inorganic contact and more complex transport pathways raise the impedance.^27–30,32,38,39^

Temperature-dependent ionic conductivity was then used to determine whether this compositional difference persists over a broader thermally activated range.

As shown in Fig. 6, the ionic conductivity of every sample increases continuously with temperature, demonstrating a stable thermally activated relationship between ion migration and local polymer/ionic-liquid dynamics. A clear conductivity hierarchy is retained throughout the tested temperature range: pristine PPC exhibits the lowest conductivity; PYR_13_TFSI incorporation increases it substantially; Zn(TFSI)_2_ addition increases it further; and the quaternary system consistently maintains the highest conductivity.^23,27,28,33,37^

**Fig. 6:**
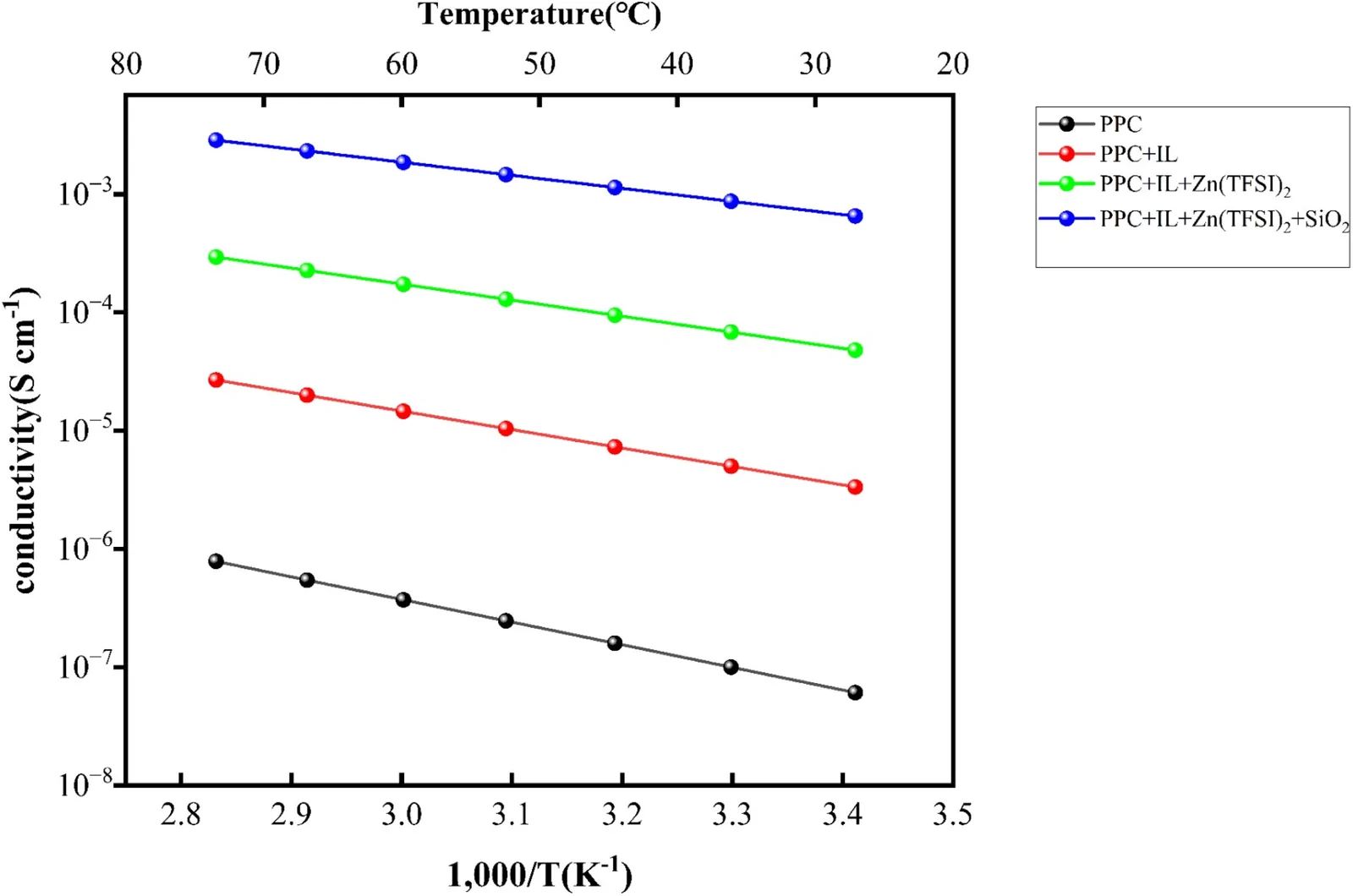
Temperature-dependent ionic conductivities of PPC-based composite electrolytes. Conductivities were calculated from EIS measurements in SS|electrolyte|SS cells after stabilization at each temperature from 20 to 80 °C (10^6^–0.01 Hz).

**Fig. 7:**
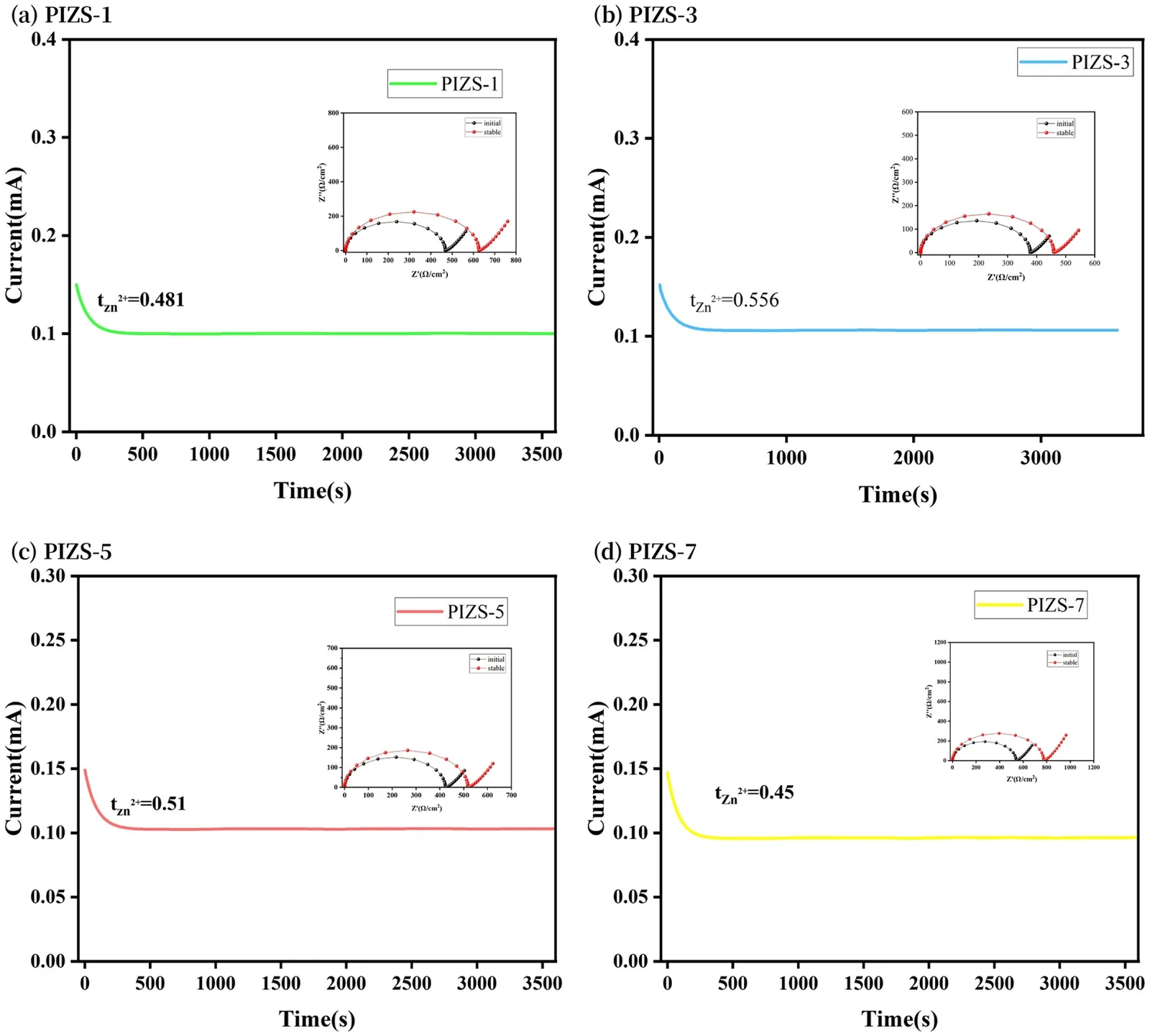
Apparent Zn^2+^ transference-number results for PIZS-*x* composite electrolytes with different SiO_2_ contents: (a) PIZS-1; (b) PIZS-3; (c) PIZS-5; and (d) PIZS-7. Values were obtained from dc polarization combined with EIS using Zn|electrolyte|Zn symmetric cells.

Total ionic conductivity contains contributions from multiple mobile species, including Zn^2+^, PYR_13_^+^, and TFSI^−^. Dc polarization combined with EIS was therefore further used to compare Zn^2+^-associated steady-state responses at different SiO_2_ contents.^23,26–28^

The apparent Zn^2+^ transference numbers of PIZS-1, PIZS-3, PIZS-5, and PIZS-7 are 0.481, 0.556, 0.510, and 0.450, respectively, exhibiting a composition dependence that first increases and then decreases. PIZS-3 reaches the highest value, 0.556, among the tested samples, consistent with the lowest-impedance composition in Fig. 5. For this multicarrier system, the parameter compares the relative contribution of the Zn^2+^-associated steady-state current under identical test conditions. Its maximum coincides with the low-impedance position at 3 wt% SiO_2_, demonstrating a consistent composition-dependent response under the present test conditions. Because PIZS-9 was not subjected to dc-polarization testing, the transference-number comparison is limited to the 1–7 wt% SiO_2_ range.^26–28^

Within the tested property set and composition range, PIZS-3 can therefore be identified as a favorable window for bulk transport, combining low bulk resistance, high room-temperature ionic conductivity, and the highest apparent Zn^2+^ transference number. The following section examines whether these transport characteristics correspond to the interfacial response during repeated Zn plating/stripping.

### 3.3 Correlation between the bulk-transport metrics of PIZS-3 and Zn–metal interfacial response

Zn|electrolyte|Zn symmetric cells were used to evaluate polarization changes during Zn plating/stripping. Fig. 8 presents stepwise current-density responses at 0.5–5.0 mA cm^−2^ and 1 mAh cm^−2^ per half-cycle, whereas Fig. 9 presents long-term cycling at 1 mA cm^−2^ and 1 mAh cm^−2^ per half-cycle (1 h per half-cycle). These tests probe interfacial response over a broad current-density range and sustained plating/stripping stability, respectively, thereby jointly reflecting electrolyte regulation of the Zn–metal interface.

**Fig. 8:**
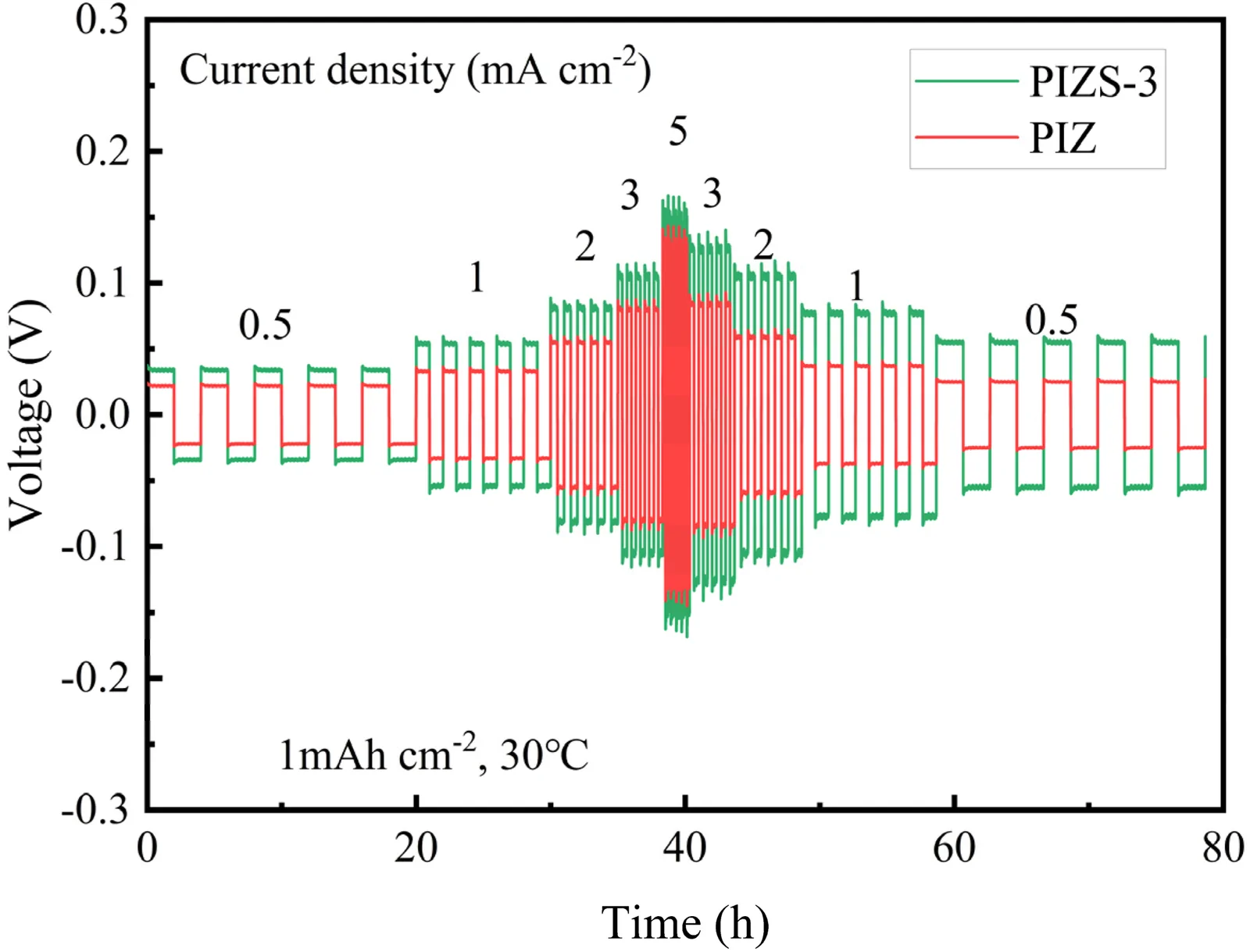
Stepwise-current-density Zn plating/stripping profiles of Zn|PIZ|Zn and Zn|PIZS-3|Zn symmetric cells at 30 °C, 0.5–5.0 mA cm^−2^, and 1 mAh cm^−2^ per half-cycle. The current-density sequence was 0.5, 1, 2, 3, 5, 3, 2, 1, and 0.5 mA cm^−2^.

**Fig. 9:**
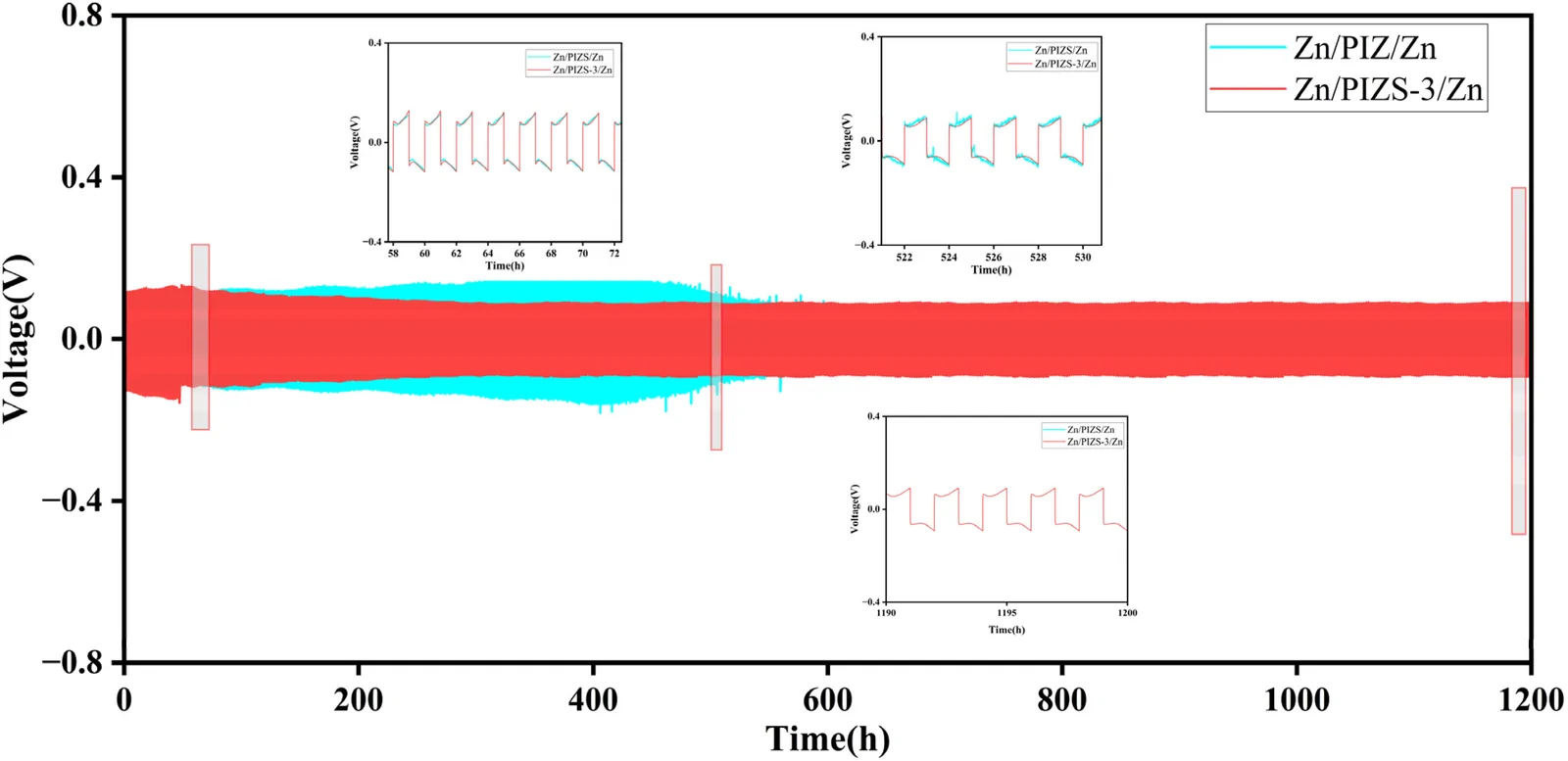
Long-term Zn plating/stripping cycling performance of Zn|PIZ|Zn and Zn|PIZS-3|Zn symmetric cells at 1 mA cm^−2^ and 1 mAh cm^−2^ per half-cycle. Each half-cycle lasted 1 h.

Fig. 8 shows that Zn|PIZ|Zn and Zn|PIZS-3|Zn maintain clear alternating plating/stripping voltage responses during the stepped sequence of 0.5, 1, 2, 3, 5, 3, 2, 1, and 0.5 mA cm^−2^. The voltage amplitude increases with current density and decreases correspondingly as the current returns to lower values. Both electrolytes exhibit reversible plating/stripping behavior at 5.0 mA cm^−2^ and 1 mAh cm^−2^. The PIZS-3 polarization response displays a current-density dependence distinct from that of PIZ, demonstrating effective SiO_2_-mediated regulation of high-rate interfacial processes.

The long-term cycling results in Fig. 9 show that, at 1 mA cm^−2^ and 1 mAh cm^−2^ per half-cycle (1 h per half-cycle), PIZS-3 exhibits smaller voltage fluctuations and lower polarization than PIZ. Zn|PIZ|Zn develops pronounced voltage fluctuations and polarization after approximately 550 h, whereas the alternating plating/stripping voltage response of Zn|PIZS-3|Zn remains stable for more than 1200 h. Together with Fig. 5 and 7, these results show that the low bulk resistance, higher Zn^2+^-associated steady-state contribution, and favorable long-term Zn|Zn response are simultaneously achieved at the PIZS-3 composition, demonstrating that an appropriate SiO_2_ content effectively optimizes both bulk transport and the Zn–metal interface.

### 3.4 Full-cell response and target-dependent composition windows

Symmetric cells primarily probe the Zn–metal interface, whereas V_2_O_5_//Zn full cells additionally integrate cathode-reaction kinetics, transmembrane ion transport, and interfacial processes at both electrodes. Full cells were therefore used to assess the behavior of the PIZS-3 composition under more complex operating conditions and to determine whether different target properties favor the same composition window.

Fig. 10a reveals a composition trend distinct from that of bulk transport. Within the tested SiO_2_ range of 0–7 wt%, the discharge capacities at all rates follow the order PIZS-7 > PIZS-5 > PIZS-3 > PIZS-1 > PIZ. For PIZS-3, the discharge capacities at 0.1, 0.2, 0.5, 1, and 2 C are approximately 258, 247, 229, 196, and 161 mAh g^−1^, respectively, and recover to approximately 250 mAh g^−1^ after returning to 0.1 C. PIZS-7 maintains higher capacity at every corresponding rate and still delivers approximately 190 mAh g^−1^ at 2 C. Thus, PIZS-3 is not the highest-capacity composition in the present full-cell rate test; within the tested 0–7 wt% range, the optimum rate-capacity position shifts to PIZS-7.

**Fig. 10:**
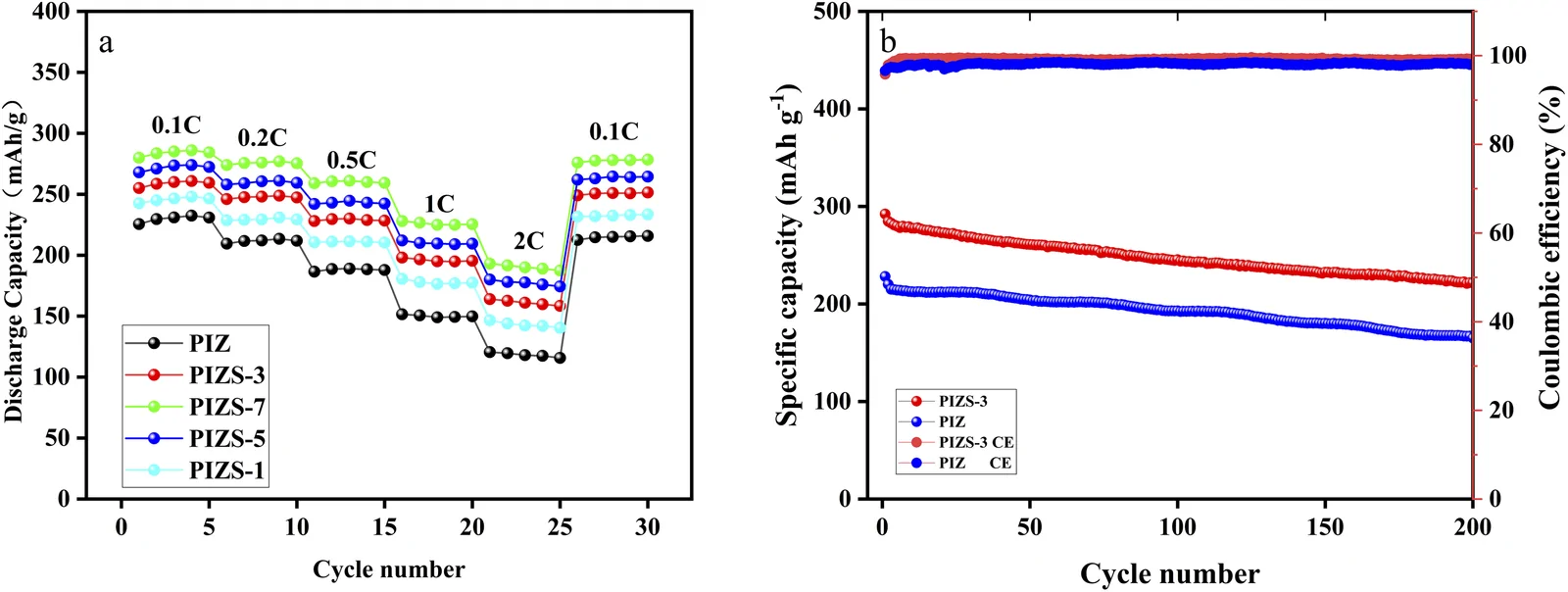
Full-cell performance of V_2_O_5_|electrolyte|Zn cells: (a) rate performance with electrolytes containing different SiO_2_ contents; and (b) comparison of cycling performance between V_2_O_5_|PIZ|Zn and V_2_O_5_|PIZS-3|Zn. PIZ denotes the SiO_2_-free reference sample in both panels. Rate testing in (a) was performed at 0.1, 0.2, 0.5, 1, and 2 C, followed by 0.1 C, within a 1.5 V voltage window.

Fig. 5, 7 and 9 collectively show that 3 wt% SiO_2_ lowers bulk impedance, enhances Zn^2+^-associated steady-state transport, and stabilizes the Zn–metal interface. The capacity of a complete V_2_O_5_ full cell is additionally governed by cathode reactions, electrolyte/cathode contact, and interfacial processes at both electrodes, resulting in more complex kinetic synergy. The full-cell rate capacity reaches its maximum at PIZS-7 within the tested composition range, indicating that increasing SiO_2_ content favors high-rate capacity in the full cell.

Fig. 10b further substantiates the beneficial effect of PIZS-3 on full-cell cycling performance. V_2_O_5_|PIZS-3|Zn retains a discharge capacity of approximately 225 mAh g^−1^ after 200 cycles, markedly higher than that of V_2_O_5_|PIZ|Zn, while the coulombic efficiency remains close to 100%. The cycling advantage of PIZS-3 and the rate-capacity advantage of PIZS-7 together indicate that different SiO_2_ contents optimize bulk transport, the Zn–metal interface, and high-rate full-cell processes to different extents, thereby defining composition windows for distinct performance targets.

These results provide a basis for extending the PIZ/PIZS electrolytes to thicker V_2_O_5_ electrodes. Increasing the active-material loading would raise the areal capacity; at low rates, the capacity could increase nearly proportionally as active-material utilization remains high. At higher rates, however, the longer Zn^2+^ diffusion path and increased electronic/ionic resistance would amplify concentration polarization, widen the charge/discharge voltage gap, and reduce specific capacity and capacity retention. Under these conditions, electrode-level transport and electrolyte/cathode contact would play a greater role, and the composition delivering the best rate capacity may therefore differ from PIZS-7 identified in the present study. High-loading electrodes can be developed by optimizing coating thickness, electrode porosity, conductive-agent/binder ratio, electrolyte uptake, and calendaring. Future evaluations should therefore emphasize areal capacity and areal current density alongside gravimetric capacity to establish how the composition–property relationship translates to practical electrode architectures.

### 3.5 Multiscale composition–property correlations of SiO_2_ content: brief summary

Collectively, Fig. 1–10 establish multiscale composition–property correlations for SiO_2_ content in a fixed PPC/PYR_13_TFSI/Zn(TFSI)_2_ matrix. SiO_2_ incorporation regulates the local chemical environments associated with PPC oxygen-containing groups, TFSI^−^, and Zn salt. At 3 wt% SiO_2_, the electrolyte exhibits the lowest impedance, a room-temperature ionic conductivity of approximately 6.0 × 10^−4^ S cm^−1^, the highest apparent Zn^2+^ transference number, and favorable long-term Zn|Zn cycling response. PIZS-7, in contrast, delivers the highest capacity in full-cell rate tests over the 0–7 wt% SiO_2_ range.

Accordingly, *ca.* 3 wt% SiO_2_ is the preferred composition window for bulk transport and Zn–metal interfacial response, whereas PIZS-7 is more suitable for maximizing full-cell rate capacity within the tested range. The advantages of different SiO_2_ contents at different performance scales demonstrate that filler loading should be optimized for the target function.

### 3.6 Post cycling Zn electrode morphology and interfacial stability

To directly examine the Zn/electrolyte interface after prolonged plating/stripping, the Zn electrodes were recovered after cycling and characterized by SEM. As shown in Fig. 11a, the pristine Zn foil has a relatively smooth surface with only shallow rolling marks and local defects. This morphology provides the reference state for evaluating the cycling-induced surface evolution.

**Fig. 11:**
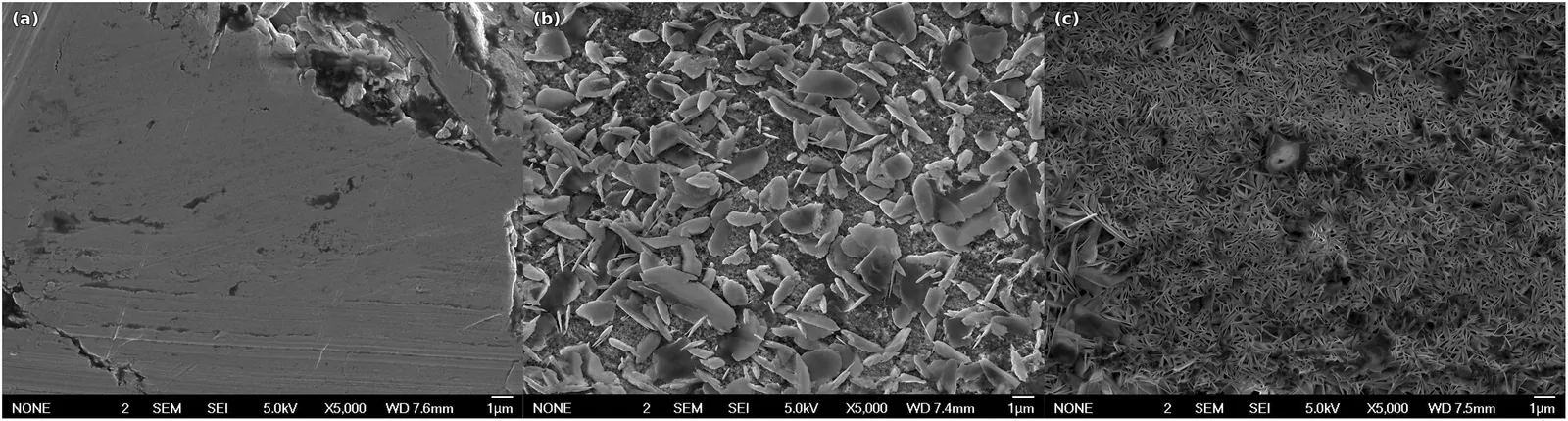
SEM images of Zn electrodes: (a) pristine Zn foil; (b) Zn electrode after more than 1200 h of cycling at 1 mA cm^−2^ and 1 mAh cm^−2^ per half-cycle in the Zn|PIZS-3|Zn cell; and (c) Zn electrode after cycling under the same conditions in the Zn|PIZ|Zn cell. All images were recorded at 5000×.

After more than 1200 h of cycling in the Zn|PIZS-3|Zn cell, the Zn electrode displays a comparatively compact lamellar/particulate deposition layer without obvious high-aspect-ratio needle-like protrusions (Fig. 11b). No large isolated Zn agglomerates are apparent at the SEM observation scale. In contrast, the Zn electrode paired with the PIZ electrolyte develops a loose and highly heterogeneous surface with abundant needle-like deposits (Fig. 11c), indicating localized Zn^2+^ flux and non-uniform plating. The compact morphology observed for PIZS-3 is consistent with its lower polarization and stable long-term cycling response in Fig. 9. These results indicate that the SiO_2_-regulated local ionic environment promotes more homogeneous Zn deposition, suppresses apparent dendritic growth, and limits obvious accumulation of electrically isolated Zn during cycling.

## 4 Conclusions

This work establishes clear relationships between SiO_2_ content and local spectroscopic response, bulk transport, Zn–metal interfacial response, and full-cell performance in a fixed PPC/PYR_13_TFSI/Zn(TFSI)_2_ matrix. XRD indicates that the composite electrolytes remain predominantly amorphous, with no detectable long-range crystalline order, while FTIR and peak-fitted O 1s/XPS data are consistent with SiO_2_-induced changes in the local oxygen-containing coordination environment that are favorable for ion transport. The use of CO_2_ as a comonomer-derived carbon source for PPC also gives this electrolyte platform a CO_2_-valorization-oriented materials basis.

A SiO_2_ content of *ca.* 3 wt% corresponds to low impedance, a room-temperature ionic conductivity of approximately 6.0 × 10^−4^ S cm^−1^, the highest apparent Zn^2+^ transference number, and favorable Zn|Zn interfacial response. In stepwise tests at 0.5–5.0 mA cm^−2^ and 1 mAh cm^−2^ per half-cycle, both electrolytes maintain reversible Zn plating/stripping behavior. During long-term cycling at 1 mA cm^−2^ and 1 mAh cm^−2^ per half-cycle, PIZS-3 exhibits a stable Zn plating/stripping response. Within the 0–7 wt% SiO_2_ range, PIZS-7 delivers the highest capacity in rate testing.

Filler-content selection should therefore be guided by the target function and evaluation scale: 3 wt% SiO_2_ optimizes bulk transport and the Zn–metal interface, whereas higher SiO_2_ contents improve full-cell rate capacity. Post-cycling SEM further confirms this interfacial regulation: after 1200 h of cycling, PIZS-3 produces a relatively compact Zn deposition layer without apparent needle-like dendrites, whereas the PIZ reference yields a loose surface covered with needle-like Zn deposits.

## Author contributions

All authors contributed to the conception and design of the study, the experimental work, data analysis, and preparation or revision of the manuscript, and approved the final version.

## Conflicts of interest

The authors declare that they have no known competing financial interests or personal relationships that could have appeared to influence the work reported in this study.

## Data Availability

The data supporting the findings of this study are available from the corresponding author upon reasonable request.
